# Differential cued recall memory impairment in mild cognitive impairment due to Alzheimer's disease versus Parkinson's disease

**DOI:** 10.1111/jnp.70010

**Published:** 2025-08-28

**Authors:** Ondrej Bezdicek, Jiří Motýl, Tomáš Nikolai, Adéla Fendrych Mazancová, Jakub Hort, Robert Jech, Martin Vyhnálek, Hana Horáková

**Affiliations:** ^1^ Department of Neurology and Centre of Clinical Neuroscience First Faculty of Medicine and General University Hospital in Prague, Charles University in Prague Prague Czech Republic; ^2^ Faculty of Arts, Department of Psychology Charles University Prague Czech Republic; ^3^ School of Psychology University of New York in Prague Prague Czech Republic; ^4^ Memory Clinic, Department of Neurology Second Faculty of Medicine, Charles University and Motol University Hospital Prague Czech Republic; ^5^ Department of Clinical Psychology Motol University Hospital Prague Czech Republic

**Keywords:** Alzheimer's disease, cued recall, episodic memory, mild cognitive impairment, Parkinson's disease, preclinical, validity

## Abstract

Both Alzheimer's (AD) and Parkinson's disease (PD) are often associated with memory dysfunction, but their pathophysiological underpinnings differ. The current research aimed to differentiate specific profiles of memory impairment due to AD versus PD. We used controlled learning and cued recall paradigm based on the Memory Binding Test (MBT) in ‘clinically cognitively normal’ controls (CN; *n* = 161), in patients with amnestic mild cognitive impairment due to AD (AD‐aMCI; *n* = 50) and due to PD (PD‐MCI; *n* = 22), and in PD with normal cognition (*n* = 18) as based on performance in the neuropsychological battery to prevent circularity in diagnostic decision‐making. We applied analysis of covariance (ANCOVA) and Receiver Operating Characteristic (ROC) analysis to determine between‐group differences and detection potential of the MBT. We found statistically large between‐group differences with worse memory performance in paired cued recall conditions in AD‐aMCI<PD‐MCI; AD‐aMCI<PD‐NC; AD‐aMCI<CN (*p* < .001 after Bonferroni correction), and to a lesser extent in PD‐MCI<CN (*p* = .039). However, PD‐NC did not differ from PD‐MCI, and PD‐NC did not differ from CN (*p* > .050). The detection potential of MBT paired cued recall for differentiating memory impairment in AD‐aMCI from CN yielded an AUC of 90% (95% CI, 85–96) and an AUC of 91% (95% CI, 81–>99) between AD‐aMCI and PD‐MCI. Associative memory and binding impairment are most pronounced in AD‐aMCI in comparison to PD‐MCI and controls. Overall, the MBT is an efficient tool for the differential diagnosis of memory impairment due to the two most common neurodegenerative diseases.

**Key points**

- Our study contributes significant new findings of memory disorder in the two most prevalent neurodegenerative diseases: Parkinson's disease and Alzheimer's disease.
- We compared the memory profile in analogous stages of cognitive impairment, mild cognitive impairment in Parkinson's disease and Alzheimer's disease.
- The data show a pronounced associative memory deficit in Alzheimer's disease and mild cognitive impairment in comparison to mild cognitive impairment in Parkinson's disease.
- The study makes a new pathway into understanding key differences in memory impairment in these devastating neurodegenerative diseases.

## INTRODUCTION

Declarative memory is a specific memory system for conscious recollection of facts and events (Milner et al., 1998; Squire, 2004). One of the key subsystems of declarative memory is associative memory, which serves to organize our memories into memory structures according to their contiguity in time and space (Calkins, 1896; Kahana, 2014).

One of the classic experimental designs for investigating associative memory is the cued recall paradigm. It is based on learning a list of specific items, such as words, pictures, etc., with a superordinate category serving as a cue during recall. Within the associative memory system, the role of the memory binding process is to relate two pieces of information into an arbitrary combination (Mayes et al., 2007). Previous evidence has shown that the left inferior frontal gyrus supports the generation of associations between items and the hippocampus then binds these associations to form new memories (Addis & McAndrews, 2006; Cohen et al., 1999; Eichenbaum, 2001; Hannula & Ranganath, 2008; Schacter et al., 1996).

Regarding the key neurobiological structures participating in the consolidation (acquisition) of new memories, there have been attempts to translate experimental binding and cued recall paradigms into clinical research of memory disorders (Buschke, 1973, 2014; Loewenstein et al., 2018; Rentz et al., 2013; Vyhnalek et al., 2019). Memory impairment, regardless of the cause, usually manifests as low retrieval; however, low retrieval may be the result of different memory processes impairments (encoding, retention), or even of non‐memory difficulties (sensory, attentional and executive); for a review (Vyhnalek et al., 2019). To differentiate primary encoding and retrieval deficits from other non‐memory causes of low retrieval, the controlled learning and cued recall paradigms were shown to be useful since they specifically facilitate the encoding and maximize the retrieval (Buschke, 2014; Tulving & Thomson, 1973). Such a measure based on the controlled learning/cued recall paradigm is potentially efficient for differential diagnosis of memory disorders caused by different pathologies, for example, Alzheimer's disease (AD; β‐amyloidopathy) versus Parkinson's disease (PD; α‐synucleinopathy).

In AD, the pathological cascade of extracellular accumulation of amyloid‐β followed by intracellular accumulation of hyperphosphorylated tau, mainly in the medial temporal lobe (MTL), causes insidious, progressive declarative memory impairment that starts years before dementia onset (Braak & Braak, 1991; Clark et al., 2011; Jack et al., 2013). To assist clinicians in identifying those older adults at risk of developing AD, mild cognitive impairment (MCI) has been introduced as the transitional stage between cognitively unimpaired and demented due to AD (Albert et al., 2011; Jack et al., 2018; Petersen, 2004; Petersen et al., 1999).

Originally, patients with amnestic MCI due to AD (AD‐aMCI) were supposed to experience a predominant declarative memory deficit greater than expected in healthy ageing individuals, with inefficient free and cued recall, with other cognitive domains largely preserved (Bird & Luszcz, 1991; Buschke et al., 1997; Carlesimo et al., 2011; Lemos et al., 2014; Loewenstein et al., 2009; Mowrey et al., 2016; Petersen, 2007). Cued recall impairment was considered to be specific for AD‐related memory deficit (Dubois et al., 2007); however, recent findings have shown a more complex picture. Using the traditional tests, such as the Free and Cued Selective Reminding Test (FCSRT) or the Enhanced Cued Recall (ECR), the cued recall impairment followed the free recall impairment slightly later (Grober et al., 2019). It was the free recall impairment that was predictive of incident MCI (Grober et al., 2022). In addition, cued recall was not more efficient than free recall measures, exemplified by the Rey Auditory Verbal Learning Test (RAVLT), to predict dementia development.

For the limitations of standard memory tests (e.g. RAVLT), the Memory Binding Test (MBT; previously referred to as the Memory Capacity Test, MCT) was developed to detect the earliest signs of memory impairment by tapping domain‐specific rather than domain‐general processes (Buschke, 2014). In contrast to the traditional FCSRT, it relies on cued recall over free recall by providing two exemplars for each cue. Unlike the FCSRT, the MBT eliminates the ceiling effects and requires participant associative binding (Buschke, 2014; Gramunt et al., 2016). The MBT evaluates verbal memory and binding capabilities by using two lists of 16 words, each associated with 16 category cues. The test is based on the principle of encoding specificity, ensuring that both encoding and recall contexts are matched. The MBT is designed to measure cued recall, binding memory and the impact of semantic interference, making it useful for identifying preclinical memory decline (Markova et al., 2023; Vyhnalek et al., 2022, 2014).

In a similar vein, in the course of the PD pathological cascade, alpha‐synuclein accumulates in the autonomic, limbic and somatomotor systems and leads to intracerebral formation of abnormal proteinaceous Lewy bodies and neurites in the neocortex (Braak et al., 2004; Braak & Del Tredici, 2017; Del Tredici & Braak, 2016).

Current memory research in PD with mild cognitive impairment (PD‐MCI) reveals deficits in both encoding and free recall (Bezdicek et al., 2019; Bronnick et al., 2011; Chiaravalloti et al., 2014; Costa et al., 2014; Edelstyn et al., 2015; Wallace et al., 2022). These impairments are primarily due to attentional and executive dysfunction resulting from fronto‐striatal dysfunction (Bezdicek et al., 2018; Peraza et al., 2017). Additionally, PD‐MCI patients exhibit impaired associative and binding mechanisms during cued recall or associative reinstatement tasks, such as the Memory Binding Test (MBT) and Associative Reinstatement Memory (ARM) test (Bezdicek et al., 2019; Cohn et al., 2016). This impairment is linked to reduced functional connectivity between the anterior hippocampi and the precuneus and superior parietal cortex (Bezdicek et al., 2019; Bronnick et al., 2011; Buytenhuijs et al., 1994; Chiaravalloti et al., 2014; Cohn et al., 2016; Edelstyn et al., 2015; Higginson et al., 2005; McAndrews et al., 2020; Whittington et al., 2000, 2006).

Overall, AD‐aMCI and PD‐MCI are not mutually exclusive from a neuropathological point of view. Between 20% and 33% of individuals with PD also have comorbid AD pathology. Additionally, hippocampal atrophy can be present in both PD and AD. Temporal sequence is not clear, that is, if AD precedes PD or vice versa (de la Monte et al., 1989; Krajcovicova et al., 2019; Smith et al., 2019).

**FIGURE 1:**
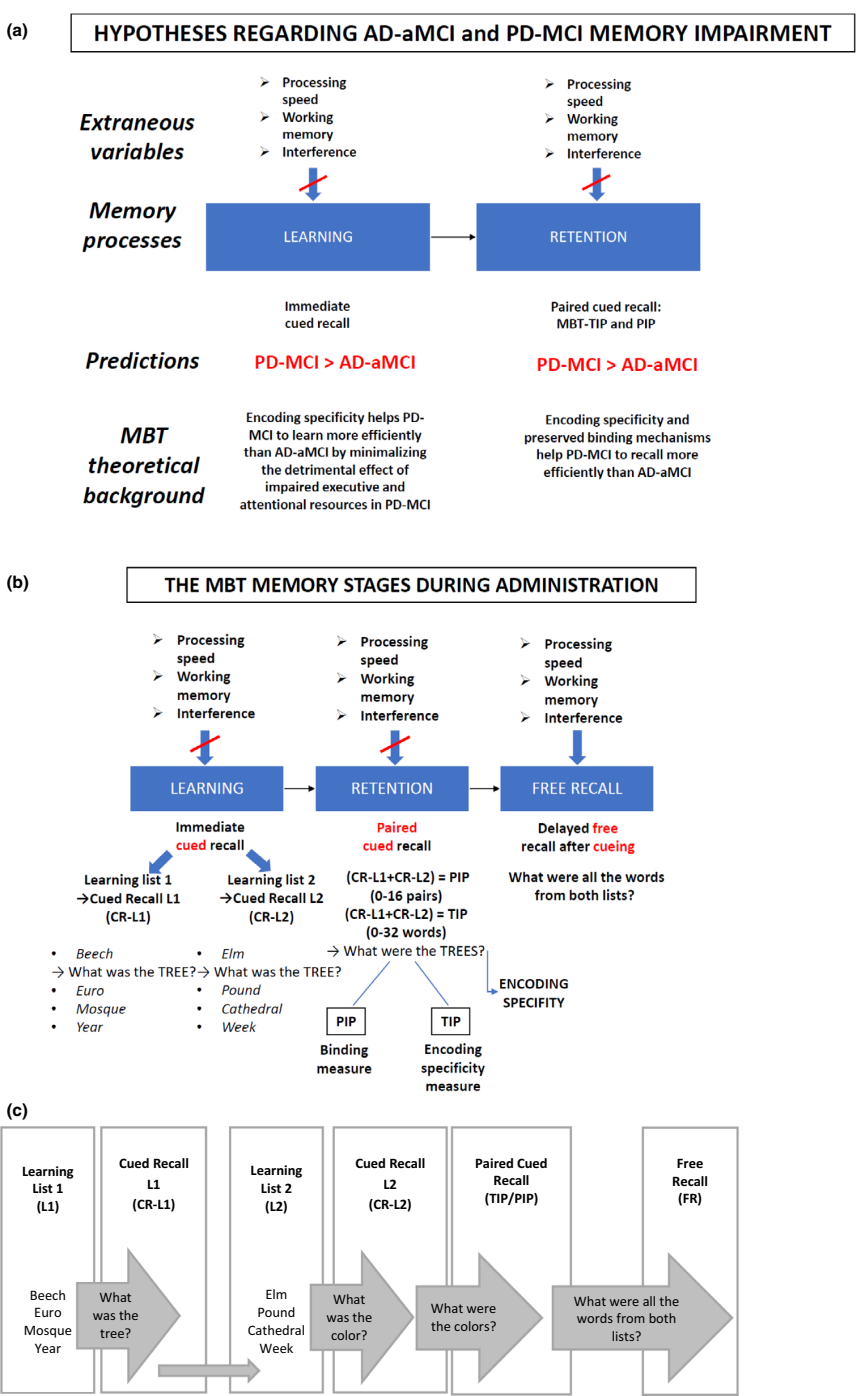
(a) Hypotheses and predictions regarding AD‐aMCI and PD‐MCI groups. (b) Memory stages during the MBT administration. (c) The administration process of the MBT according to Markova et al. (2023). In the learning phase, four‐word items are presented per page at a time, the examiner names a category (e.g. tree) and the tested individual is asked to choose the corresponding word item from the four items available (e.g. beech); after identifying all the 16 items from the List 1, the examiner verbally presents the cue alone and asks the tested individual to recall the corresponding word (CR‐L1; range 0–16). The same procedure follows for List 2 (CR‐L2; range 0–16). In the paired cued recall phase, the tested individual is presented with the semantic cues again, now he/she is asked to recall the corresponding items from both lists (TIP, the total number of items cued recalled, range 0–32; PIP, the number of pairs cued recalled; range 0–16). This is followed by the free recall phase, in which the tested individual is asked to freely recall as many word items as possible from both lists (FR).

In the current research, we put to the test the hypothesis that a neuropsychological tool, the MBT, based on the Grober‐Buschke controlled learning/cued recall and enriched by the memory binding paradigm, can differentiate between MCI due to two clinically different but neuropathologically partly overlapping neurodegenerative diseases: AD‐aMCI and PD‐MCI (Carlesimo et al., 2011; Dubois et al., 2007; McAndrews et al., 2020). More specifically, we suppose that associative binding mechanisms will be more impaired in AD‐aMCI than in PD‐MCI (for more details, see Figure 1a).

## METHOD

### Procedure

All participants were tested under standard conditions for neuropsychological assessment and were instructed in the aims and procedures of the study. All controls were matched with PD‐NC, PD‐MCI or AD‐aMCI patients based on frequency matching (by comparing the distributions of characteristics among the cases). The institutional review board of the General University Hospital and Motol University Hospital approved the study. All participants (patients and controls) signed written consent to be included in the study. All procedures conformed to the Declaration of Helsinki.

### Participants

#### AD‐aMCI sample

Participants with aMCI due to AD (*n* = 50) were recruited from the Czech Brain Aging Study (CBAS; [Sheardova et al., 2019]) at the Memory Clinic, Department of Neurology, Second Faculty of Medicine, Charles University and Motol University Hospital in Prague. They were referred to the memory clinic by general practitioners, neurologists or psychiatrists for subjective cognitive complaints reported by themselves, and/or by their close informants. All the AD‐aMCI participants met the clinical criteria outlined in recommendations from the National Institute on Aging‐Alzheimer's Association workgroups on diagnostic guidelines for AD (Albert et al., 2011), including concerns regarding a change in cognition, evidence of memory impairment, preservation of independence in activities of daily living and absence of dementia. All the AD‐aMCI individuals were neuropsychologically assessed using the Uniform Data Set (UDS‐Cz 2.0; (Nikolai et al., 2018)), enriched by additional memory, executive and visuospatial tests (RAVLT; Brief Visuospatial Memory Test‐Revised [BVMT‐R], Rey–Osterrieth Complex Figure Task, copy and reproduction condition; and Prague Stroop test), further ‘AD‐aMCI battery.’ These tests were used to diagnose the aMCI syndrome. Memory impairment was established when the patients scored ≥1.5 *SD* below the mean of age‐ and education‐adjusted norms on at least one of the following six memory variables across three memory tests: Logical Memory (LM) I immediate recall, LM I delayed recall, RAVLT total recall (sum of trials 1–5), RAVLT delayed recall, BVMT‐R total recall (sum of trials 1–3) and BVMT‐R delayed recall.

A total of 19 aMCI patients underwent CSF withdrawal, 13 underwent PET imaging, and 10 underwent both procedures. Altogether, there were 22 individuals with aMCI with a high likelihood of AD aetiology (AD‐aMCI‐HL). They met the core clinical criteria for MCI, and in addition, they had positive biomarkers for β‐amyloid and neuronal injury: that is, reduced Aβ42 and elevated cerebrospinal fluid (CSF) phosphorylated tau 181 (p‐Tau181) [<620 pg/mL and >61 pg/mL, respectively] (Gobom et al., 2022) and/or were positive for Aβ based on flutemetamol PET imaging. In patients who did not undergo CSF analysis, neuronal injury was assessed through hippocampal atrophy, determined via visual rating using the Medial Temporal Atrophy (MTA) scale. We applied the cut‐off values established by Ferreira et al. (2015), using an MTA score of ≥2 for patients aged 75 years and older, and ≥1.5 for younger patients, to indicate hippocampal atrophy, by age‐adjusted criteria (Ferreira et al., 2015).

In 28 patients, no amyloid biomarkers were available. Among them, 12 individuals with aMCI fulfilled the criteria for MCI due to AD with intermediate likelihood (they met the core clinical criteria for MCI, and in addition, they had a positive biomarker reflecting neuronal injury with an untested biomarker of β amyloid). The remaining 16 aMCI patients met only the core clinical criteria (i.e. evaluation of neuronal injury was inconclusive and evidence of β amyloid marker was not available).

#### PD sample

Patients (*N* = 40) were recruited at the Department of Neurology, General University Hospital. The clinical diagnosis of PD was confirmed by a movement disorders specialist (PD) according to the Movement Disorders Society (MDS) clinical diagnostic criteria (Postuma et al., 2015). PD symptoms were assessed with the UPDRS motor score (Part III) ‘on’ state (18.12 ± 9.74), Hoehn and Yahr staging (2.02 ± .52), and PD duration (12.03 ± 4.46 years). Levodopa equivalent (1393.83 ± 679.77) daily dose was also calculated (Jost et al., 2023; Tomlinson et al., 2010). No biomarker data were available in the PD‐MCI group, which limits our ability to assess for potential AD co‐pathology in this population.

#### PD‐MCI classification

We applied a neuropsychological assessment using a standard protocol at Level II, as recommended by the MDS for PD‐MCI (Litvan et al., 2012). The protocol consists of a cognitive assessment (further ‘PD‐MCI battery’) that contained 10 tests in five cognitive domains and was previously standardized including normative data (Bezdicek, Nikolai, et al., 2017; Bezdicek, Sulc, et al., 2017). The diagnosis of PD‐MCI was based on MDS Task Force criteria for PD‐MCI Level 2 criteria (comprehensive assessment) and required impaired performance on at least 2 of the 10 neuropsychological tests. Impaired performance on a neuropsychological test was defined as a score that was at least 2.0 SDs below the age‐adjusted mean from normative samples (Bezdicek, Sulc, et al., 2017). We classified 22 patients as PD‐NC and 18 as PD‐MCI. To prevent circularity in diagnostic decision‐making, none of the classifications (PD‐MCI or AD‐aMCI) included the MBT.

#### Clinically cognitive normal controls

The clinically cognitively normal control (CN; *N* = 161) sample was recruited from the general community through advertisements (non‐random sampling), and a brief medical history was obtained by telephone. Only those individuals who scored 26 points or higher on the Mini‐Mental State Examination (MMSE) were included.

Individuals were not included in any of the groups (AD‐aMCI, PD‐MCI, PD‐NC and CN) if they had psychotic manifestations (hallucinations or delusions), were taking anticholinergic medications, or had other medical or neurological conditions potentially leading to cognitive impairment (e.g. epileptic seizure, tumour, stroke or head trauma). They were also not included if they had a history of head trauma with loss of consciousness, cerebrovascular accident, alcohol or other psychoactive substance abuse, comorbid neurological or psychiatric diseases, or ongoing delirium. In addition, individuals were not included if they were currently undergoing radio‐ or chemotherapy, had a major medical condition (e.g. myocardial infarction and diabetes mellitus), or had sensory deficits that would hinder the neuropsychological assessment. Furthermore, individuals were not included if they suffered from severe or moderate depression according to the Beck Depression Inventory (BDI‐II) for PD‐MCI, PD‐NC or CN, and according to the Geriatric Depression Scale (GDS‐15, the 15‐item version) for the AD‐aMCI (Beck et al., 1996; Ciharova et al., 2020). Finally, in PD, patients with clinical or laboratory signs of atypical parkinsonism or PD dementia according to MDS criteria (Emre et al., 2007) were not included.

### Materials

#### Memory Binding Test

The Memory Binding Test (MBT) is a controlled learning and encoding specificity paradigm measure (Bezdicek et al., 2019; Buschke, 2014; Buschke et al., 2017; Gramunt et al., 2016; Loewenstein et al., 2018; Markova et al., 2023; Mowrey et al., 2016; Thomson & Tulving, 1970). Controlled learning minimizes the influence of sustained attention upon successful learning and the use of variable learning strategies including retrieval‐specific cues to access memory for what was learned (Buschke, 2014). The MBT involves two coordinated lists of 16 items that are based on category membership. Initially, participants learn a first list of words with category‐based cues in the first trial (e.g. Tree → Beech, designated as cued recall from List 1; CR‐L1). They are then presented with a second list of words that includes new items from the same categories (e.g. Tree → Elm, referred to as CR‐L2). This process is followed by a paired cued recall task, in which participants are required to associate the items within each category (e.g. Beech and Elm). Finally, free recall of all the items from both lists of words follows. From this technique, we can derive two essential measures: TIP (total number of items cued recalled in the paired condition, range 0–32), and PIP (the number of pairs cued recalled in the paired condition, range 0–16). The TIP can be understood as an encoding specificity measure and associative memory measure (the ability to recall both category members given the semantic cue) and the PIP as a binding measure (the ability to bind pairs of items). Beyond these measures, a measure of the total number of items recalled in the 2‐min free recall condition can be also derived (FR). See Figure 1b,c.

### Statistical analyses

All analyses were performed using R Statistical Software version 4.3.2 in the RStudio environment (R Core Team, 2022; RStudio Team, 2022). Receiver operating characteristic (ROC) curves were produced using the package pROC (Robin et al., 2011), analysis of covariance (ANCOVA) was produced using the jmv package (Selker et al., 2022), robust analysis of covariance (robust ANCOVA) was produced using the package WRS2 (Mair & Wilcox, 2020). The full reproducible code is available in Supporting Information, on GitHub and in the OSF Database (Bezdíček et al., 2024). Normality was evaluated by visual inspection of histograms, Q‐Q plots, and skew and kurtosis measures. Group differences in demographic and neuropsychological characteristics were analysed using the nonparametric Kruskal–Wallis *H*‐test for scales or the Chi‐Squared test (*χ* ^2^) for categorical variables. Effect sizes were calculated by Cramer's *V* for the Chi‐Squared test, by *ε* ^2^ for the Kruskal–Wallis H‐test and by Partial *η* ^2^ for ANCOVA. *Post hoc* pairwise comparisons between groups were calculated by Tukey's HSD (honestly significant difference) test. Kendall's correlation coefficient was used to evaluate the convergent and divergent validity, the relationship between different MBT test measures and other neuropsychological test methods. Kendall's correlation coefficient and Welch's *t*‐test were used to test the relationship of MBT measures with demographic characteristics. Analysis of covariance (ANCOVA) was used to evaluate between‐group differences in neuropsychological outcomes, accounting for age and years of education and to evaluate in detail the influence of demographic variables on MBT‐TIP variance and to compare differences in MBT‐TIP across all participant groups (CN, PD‐NC, PD‐MCI and AD‐aMCI). The homogeneity of variance was tested by Levene's test. Sensitivity, specificity and receiver operating characteristic (ROC) curves with AUC were calculated, CN sample was compared with the diagnostic classification of PD‐NC, PD‐MCI, AD‐aMCI (whole group/β‐amyloid confirmed subgroup). In case of missing data, we made the pairwise deletion (available‐case analysis) to minimize the loss of cases. The level of significance was set at *α* = .05. A *post hoc* power analysis was conducted using G*Power Version 3.1.9.6 for the analyses of covariance (Faul, 2020). The analysis, with an observed effect size (Partial *η* ^2^ = .29), an alpha level of .05 and a sample size of 250, revealed an observed power of 1.0, with a critical *F* value of 2.64.

### Results

No significant difference was found in the proportion of men and women in the clinical and control groups. However, the groups differed in the years of education (H(3) = 17.8, *p* < .001) and age (H(3) = 42.83, *p* < .001); see Table 1. Patients with AD‐aMCI were older and had more years of education than PD‐MCI. Differences between the clinical and control groups were found in years of education and age. Subsequent pairwise comparisons revealed a significantly different (older) age of the AD‐aMCI group in comparison to the other three groups (e.g. CN vs. AD‐aMCI: W = −8.851, *p* < .001), and the AD‐aMCI group differed significantly in slightly more years of education from the CN and PD‐MCI groups (e.g. CN vs. AD‐aMCI: W = −4.554, *p* < .001).

**TABLE 1:** Demographic characteristics and test scores of clinically cognitively normal controls and patients' groups.

	CN	PD‐NC	PD‐MCI	AD‐aMCI	*p*‐value	Effect size
*N*	161	22	18	50		
Sex (F/M)	85/76	10/12	8/10	24/26	.813^1^	.062^3^
Age (M, *SD*, min–max)	62.63 (±9.76; 38.00–85.00)	63.82 (±7.40; 45.00–81.00)	62.78 (±9.91; 37.00–82.00)	72.27 (±7.03; 51.00–88.00)	<.001^2^ ^,c,e,f^	.171^4^
Education (years, M, *SD*, min–max)	14.50 (±2.74; 9.00–25.00)	14.80 (±2.86; 11.00–21.00)	13.11 (±2.89; 8.00–19.00)	15.90 (±3.09; 8.00–20.00)	<.001^2^ ^,c,f^	.071^4^
PD duration (years)	–	11.12 (±5.61; 4.00–26.00)	12.86 (±2.85; 9.00–18.00)	–	.200^2^	.007^4^
UPDRS‐III ‘on’ state	–	17.00 (±10.75; 4.00–39.00)	18.93 (±8.96; 3.00–32.00)	–	.519^2^	.002^4^
Hoehn/Yahr stage	–	2.00 (±.48; 1.50–3.00)	2.04 (±.60; 1.00–3.00)	–	.744^2^	.000^4^
L‐Dopa Equivalent	–	1310.86 (±747.27; .00–2828.80)	1457.27 (±603.77; 450.00–2731.00)	–	.339^2^	.004^4^
MMSE*	28.64 (±1.14; 25.00–30.00)	28.11 (±1.49; 25.00–30.00)	26.19 (±3.17; 17.00–30.00)	27.72 (±1.44; 25.00–30.00)	<.001^5^ ^,b,c^	.112^6^
TMT‐A**	41.54 (±14.07; 20.00–88.00)	40.27 (±9.04; 20.00–55.00)	69.56 (±28.43; 34.00–131.00)	46.21 (±16.79; 25.00–82.40)	<.001^5^ ^,b,d,f^	.130^6^
TMT‐B**	93.19 (±38.23; 38.00–290.00)	100.50 (±26.89; 52.00–164.00)	228.78 (±104.06; 91.00–416.00)	142.94 (±79.57; 48.80–315.00)	<.001^5^ ^,b,c,d,f^	.265^6^
PST‐D**	14.88 (±3.87; 10.00–28.00)	12.73 (±2.07; 9.00–17.00)	15.00 (±3.77; 10.00–25.00)	15.05 (±4.20; 10.39–31.97)	.030^5^ ^,a,e^	.036^6^
PST‐W**	19.41 (±6.79; 11.00–46.20)	15.18 (±2.48; 11.00–20.00)	20.61 (±5.00; 14.00–29.00)	20.38 (±14.80; 12.57–120.00)	.005^5^ ^,a,d,e^	.051^6^
PST‐C**	28.50 (±8.20; 16.40–51.00)	29.41 (±8.10; 18.00–57.00)	45.22 (±23.58; 18.00–110.00)	38.01 (±13.18; 18.00–77.90)	<.001^5^ ^,b,c,d^	.126^6^
RAVLT T1‐5	47.38 (±8.24; 28.00–74.00)	41.41 (±7.79; 28.00–56.00)	37.50 (±13.25; 15.00–64.00)	34.92 (±8.97; 18.00–55.00)	<.001^5^ ^,a,b,c^	.224^6^
RAVLT‐DR	9.14 (±2.70; 2.00–15.00)	8.00 (±2.60; 3.00–14.00)	5.94 (±3.86; .00–12.00)	3.70 (±3.36; .00–11.00)	<.001^5^ ^,b,c,e,f^	.319^6^
MBT CR L1*	15.49 (±.86; 12.00–16.00)	15.00 (±1.15; 13.00–16.00)	14.56 (±1.59; 10.00–16.00)	12.36 (±3.26; 5.00–16.00)	<.001^5^ ^,b,c,e,f^	.269^6^
MBT CR L2*	13.29 (±2.20; 7.00–16.00)	12.89 (±1.82; 9.00–16.00)	11.12 (±3.14; 4.00–16.00)	8.10 (±3.55; 2.00–14.00)	<.001^5^ ^,b,c,e^	.214^6^
MBT CR L1 + L2*	28.78 (±2.62; 22.00–32.00)	27.89 (±2.23; 24.00–31.00)	25.69 (±4.53; 14.00–32.00)	20.46 (±6.13; 9.00–30.00)	<.001^5^ ^,b,c,e^	.256^6^
MBT‐TIP*	27.96 (±3.03; 20.00–32.00)	27.16 (±2.81; 23.00–31.00)	24.56 (±5.45; 9.00–30.00)	18.02 (±7.17; 4.00–31.00)	<.001^5^ ^,b,c,e,f^	.293^6^
MBT‐PIP	12.33 (±2.76; 5.00–16.00)	11.58 (±2.61; 7.00–15.00)	9.44 (±4.07; .00–14.00)	5.51 (±3.95; .00–15.00)	<.001^5^ ^,b,c,e,f^	.404^6^
MBT‐FR 2 min	17.57 (±4.55; 6.00–28.00)	15.47 (±3.41; 8.00–24.00)	10.38 (±6.66; .00–20.00)	7.74 (±5.61; .00–18.00)	<.001^5^ ^,b,c,d,e^	.376^6^

Abbreviations: AD‐aMCI, Alzheimer's Disease Amnestic Mild Cognitive Impairment; CN, clinically cognitively normal controls; CR‐L1, Number of items cued recalled from List 1 on the MBT; CR‐L2, number of items cued recalled from List 2 on the MBT; CR L1 + L2, number of items cued recalled from List 1 and List 2 on the MBT; FR 2 min, total number of items recalled in the 2 min free recall condition on the MBT; MBT, Memory Binding Test; MMSE, Mini Mental State Examination; PD‐MCI, Parkinson's Disease Mild Cognitive Impairment; PD‐NC, Parkinson's Disease Normal Cognition; PIP, the number of pairs cued recalled in the paired condition of MBT; PST, Prague Stroop Test; PST‐C, colours; interference condition; PST‐D, dots, naming colours; PST‐W, words, weak interference; RAVLT, Rey Auditory Verbal Learning Test (RAVLT T1‐5 is the sum of all correct responses given over the five consecutive trials: (T1 + T2 + T3 + T4 + T5); RAVLT‐DR (30 min): The total number of correct words recalled on the delayed recall); TIP, total number of items cued recalled in the Paired condition on the MBT; TMT, Trail Making Test.

^1^ Chi‐squared test.

^2^ Kruskal–Wallis *H*‐test.

^3^ Crammer's V.

^4^ *ε* ^2^.

^5^ ANCOVA with Age and Years of Education as covariates.

^6^ Partial *η* ^2^; Significant between‐group differences in *post hoc* tests using Tukey's HSD (honestly significant difference) test: ^a^CN versus PD‐NC, ^b^CN versus PD‐MCI, ^c^CN versus AD‐aMCI, ^d^PD‐NC versus PD‐MCI, ^e^PD‐NC versus AD‐aMCI and ^f^PD‐MCI versus AD‐aMCI.

* The variable was log10 transformed for the purposes of ANCOVA analyses.

** The variable was reflected and log10 transformed for the purposes of ANCOVA analyses.

Between‐group differences (M, *SD*, min–max) of the main neuropsychological tests' outcomes are shown in Table 1. All tests showed significant between‐group differences, with the smallest differences in PST‐D (naming colours). *Post hoc* pairwise comparisons of the main MBT measures showed the ability of MBT to detect between‐group differences in CN and both PD‐MCI and AD‐aMCI. Additionally, two cued recall MBT conditions (PIP and TIP) differentiated between PD‐MCI and AD‐aMCI: Total number of items cued recalled in the paired condition on the MBT (MBT‐TIP: *t* = −3.00, *p‐Tukey* = .016); and the number of pairs cued recalled in the paired condition of MBT (MBT‐PIP: *t* = 3.33, *p‐Tukey* = .006).

The relationship between the main MBT indices and demographic variables was assessed in CN. There was a highly significant medium to low negative relationship between age and most MBT outcomes, but almost no significant relationship between years of education or sex and MBT measures (Table 2).

**TABLE 2:** Testing the influence of demographic characteristics on selected MBT measures in cognitively normal controls (CN).

	Age^a^	Years of education^a^	Sex^b^
MBT CR L1	−.195*	.055	2.912**
MBT CR L2	−.284***	.095	.667
MBT CR L1 + L2	−.287***	.091	1.511
MBT‐TIP	−.173*	.163*	2.272*
MBT‐PIP	−.272	.073	.774
MBT‐FR 2 min	−.271**	.092	.965

Abbreviations: CR‐L1, number of items cued recalled from List 1 on the MBT; CR‐L2, number of items cued recalled from List 2 on the MBT; CR L1 + L2, number of items cued recalled from List 1 and List 2 on the MBT; FR 2 min, total number of items recalled in the 2 min free recall condition on the MBT; MBT, memory binding test; PIP, the number of pairs cued recalled in the paired condition of MBT; TIP, total number of items cued recalled in the paired condition on the MBT.

^a^ Kendall's *tau‐b* correlation.

^b^ Welch's two‐sample *t*‐test.

* *p* < .05.

** *p* < .01.

*** *p* < .001.

As the age of participants was found to be related to MBT outcomes and the AD‐aMCI group was found to be significantly older and more educated in comparison with the other groups, we have conducted a one‐way ANCOVA with the MBT‐TIP (taken as a key memory outcome from the MBT) as a dependent variable, group membership (CN, PD‐NC, PD‐MCI and AD‐aMCI) as a fixed factor and age and years of education as covariates. We found an effect of group membership on the MBT‐TIP score, *F*(3,230) = 52.49, *p* < .001, *partial η* ^2^ = .406. Both covariates were also significantly related to the MBT‐TIP score; however, in comparison with the group membership with a much lower proportion of explained variance: Age, *F*(1, 230) = 10.39, *p* = .002, *partial η* ^2^ = .043; Education, *F*(1, 230) = 11.10, *p* = .001, *partial η* ^2^ = .046. As the assumption of homogeneity of variance for ANCOVA was violated, *F*(3, 232) = 37.9, *p* < .001, we validated the main finding that age is only a secondary explanation for the MBT‐TIP score differences between the CN and AD‐aMCI groups by running robust ANCOVA. Results from the robust ANCOVA confirmed the findings (Table S1). Kendall's tau‐b correlation was conducted to check the relationship between MBT scales and other neuropsychological tests (Table S2).

We determined the detection potential of the MBT in differentiating AD‐aMCI from CN and AD‐aMCI from PD‐MCI. A ROC curve analysis yielded an AUC of 90% (95% CI, 85–96) for MBT‐TIP and an AUC of 91% (95% CI, 85–96) for MBT‐PIP (CN vs. AD‐aMCI). These results were comparable to the discriminative validity of RAVLT Delayed Recall (AUC = 89%; 95% CI: 82–95) and slightly better than RAVLT 1–5 (AUC = 85%; 95% CI: 78–91). When assessing the potential of MBI‐TIP and PIP to discriminate between AD‐aMCI and PD‐MCI, the AUC was 76% (95% CI, 63–90) and 76% (95% CI, 62–91), respectively, suggesting that the main MBT outcomes provide only acceptable discrimination between the groups and are comparable to RAVLT Delayed Recall (AUC = 67%; 95% CI: 53–82).

The same analysis was done to differentiate the biomarker‐defined AD‐aMCI‐HL subgroup from the CN and PD‐MCI groups. A ROC curve analysis yielded an AUC of 99% (95% CI, 97–>99) for MBT‐TIP and an AUC of 98% (95% CI, 95–>99) for MBT‐PIP (CN vs. AD‐aMCI‐HL). These results were comparable to the discriminative validity of RAVLT Delayed Recall (AUC = 98%; 95% CI: 96–>99) and RAVLT 1 to 5 (AUC = 90%; 95% CI: 83–96).

Both MBT‐TIP with an AUC of 91% (95% CI, 81–>99) and MBT‐PIP with an AUC of 88% (95% CI, 76–>99) showed excellent discriminative validity between the AD‐aMCI‐HL subgroup and PD‐MCI, comparable to RAVLT Delayed Recall (AUC = 81%; 95% CI: 67–95); see Table 3. For analysis of matched samples, see Suppl. Material.

**TABLE 3:** AUCs with 95% confidence intervals for all four analyses, with the additional sub‐analyses of the AD‐aMCI‐HL subgroup.

	CN versus AD‐aMCI	CN versus AD‐aMCI‐HL	PD‐MCI versus AD‐aMCI	PD‐MCI versus AD‐aMCI‐HL	CN versus PD‐MCI	PD‐NC versus PD‐MCI
MBT‐TIP	.904 (.850–.957)	.985 (.968–>.999)	.764 (.629–.900)	.911 (.807–>.999)	.713 (.577–.848)	.643 (.452–.835)
MBT‐PIP	.905 (.846–.964)	.978 (.952–>.999)	.761 (.615–.907)	.882 (.760–>.999)	.729 (.592–.865)	.656 (.471–.841)
RAVLT 1–5	.847 (.780–.913)	.898 (.834–.961)	.553 (.379–.727)	.585 (.395–.774)	.728 (.566–.889)	.614 (.421–.806)
RAVLT‐DR	.885 (.824–.946)	.980 (.958–>.999)	.674 (.526–.823)	.808 (.667–.949)	.740 (.596–.885)	.655 (.468–.843)

Abbreviations: AD‐aMCI‐HL, Amnestic Mild Cognitive Impairment due to Alzheimer's Disease ‐ subgroup of patients with positive biomarkers for β amyloid; AD‐aMCI, Amnestic Mild Cognitive Impairment due to Alzheimer's Disease (whole group); CN, Clinically cognitively normal controls; MBT, Memory Binding Test; Number of items cued recalled from List 1 and List 2 on the MBT; PD‐NC, Parkinson's Disease Normal Cognition; PD‐MCI, Mild Cognitive Impairment due to Parkinson's Disease; PIP, The number of pairs cued recalled in the paired condition of MBT; RAVLT, Rey Auditory Verbal Learning Test (RAVLT T1‐5 is the sum of all correct responses given over the five consecutive trials: (T1 + T2 + T3 + T4 + T5); RAVLT‐DR (30 min): The total number of correct words recalled on the delayed recall); TIP, Total number of items cued recalled in the Paired condition on the MBT.

## DISCUSSION

As the diagnosis of neurodegenerative diseases progresses to increasingly earlier stages, there is a growing emphasis on detecting the most subtle alterations in memory function. This shift underscores a critical need for the development of novel memory assessment tools grounded in translational research within cognitive neuroscience. (Buschke, 2014; Loewenstein et al., 2018; McAndrews et al., 2020; Rentz et al., 2013). However, these measures require validation in large clinical samples defined based on biomarkers to determine their differential role in the detection of memory deficits due to different clinical stages of these neurodegenerative diseases (Jutten et al., 2021).

In the current study, our primary goal was to test the cued recall associative memory (as represented by MBT‐TIP) and memory binding (MBT‐PIP) in AD‐aMCI versus PD‐MCI. In general, a specific neuropsychological tool should possess the capacity to discriminate among similar diagnostic classes (endophenotypes such as MCI) in different, although partly overlapping, pathophysiological spectra (Alzheimer's amyloidopathy vs. Parkinson's synucleinopathy). More specifically, we tested the hypothesis that associative memory in AD‐aMCI‐HL in comparison to PD‐MCI (as represented by the MBT‐TIP and PIP) will be impaired to a higher degree. In AD‐aMCI, the causes are hippocampal atrophy that correlates with the tau accumulation, whereas in PD‐MCI, it is typically attributed to a mix of attentional/executive dysfunction due to dopaminergic frontostriatal disruption in the frontal lobes, and concomitantly to a cued recall deficit stemming from lower connectivity between the anterior hippocampi and the precuneus/superior parietal cortex (Bezdicek et al., 2018; Buschke et al., 2017; Cohn et al., 2016; Edelstyn et al., 2007, 2015; Markova et al., 2023; Papp et al., 2015).

We found large statistical differences and very high classification accuracy (an AUC of 91%) in the MBT‐TIP for AD‐aMCI‐HL versus PD‐MCI. Further, we found high classification accuracy (an AUC of 88%) in the MBT‐PIP for AD‐aMCI‐HL versus PD‐MCI. Overall, the data indicate that AD‐aMCI‐HL presents with pronounced cued recall, associative memory and binding deficit in comparison with PD‐MCI. These differences were accompanied by large effect sizes in both key associative memory measures: MBT‐TIP and PIP. Still, the RAVLT‐DR score also showed high classification accuracy (an AUC of 81%), unlike the RAVLT 1–5 learning score (an AUC of 59%). Regarding the evidence that PD‐MCI showed also associative binding deficits in the previous research (Bezdicek et al., 2019; Cohn et al., 2016; Edelstyn et al., 2015), we explain these clear‐cut findings of PD‐MCI>AD‐aMCI>AD‐aMCI‐HL by a precipitous decline in associative binding in AD‐aMCI‐HL. First, the key role in AD‐aMCI‐HL versus PD‐MCI may be played by the hippocampus, which is affected in PD in later stages of the disease progression. According to Braak in stage 4, alpha‐synuclein reaches the hippocampus, inducing cognitive deficits (Dickson et al., 1994; Villar‐Conde et al., 2021). Not every participant in the PD‐MCI group may have been at this or a later stage. Second, early structural and functional deficits in CA1‐3 subfields of the hippocampus, critical for associative memory, were observed in aMCI due to AD (Apostolova et al., 2010; Bartsch et al., 2011; Braak & Braak, 1991; de Flores et al., 2015; Ji & Maren, 2008; La Joie et al., 2013).

In support of these findings, the cued‐recall associative memory deficit was also more pronounced in AD‐aMCI compared to PD‐MCI. The classification accuracy was lower but still acceptable for the discrimination between the groups (an AUC of 76% for both MBT‐TIP and PIP). The classification accuracy of RAVLT 1 to 5 and DR scores was not acceptable (an AUC of 55% and 67%, respectively). In sum, our results indicate that the MBT‐TIP and PIP are superior to RAVLT‐DR and learning in classification accuracy and discriminative validity, showing a more pronounced associative memory and binding deficit in AD‐aMCI in comparison to PD‐MCI.

The results partially differ from previous research in which tests based on free and cued recall paradigms (RAVLT and Rey Osterrieth Complex Figure Test as a free recall measure; and Enhanced Cued Recall test, ECR, as a cued recall measure) have shown approximately similar correlations with hippocampal volumes (HVs) in a sample of non‐demented older adults, and similar predictive validity for conversion to dementia in a sample of aMCI patients (Vyhnalek et al., 2014, 2022). However, the results may have been influenced by the ceiling effect in the ECR (Vyhnalek et al., 2014), which is not present in the MBT (Gramunt et al., 2016). Also, we found low correlations between RAVLT 1 to 5 and MBT‐TIP and PIP; however, medium correlations of RAVLT‐DR and MBT‐TIP and PIP, which may be indicative of MBT indices being more dependent on hippocampal integrity (Table S2). The results are in line with the previous research in which performance on the MBT free and paired‐cued recall measures in both the immediate and delayed recall conditions was associated with lower HV in both subjective cognitive decline and AD‐aMCI participants, whereas only performance on the delayed‐free recall measures in standard memory tests was associated with lower HV (Markova et al., 2023). In the current study, the MBT had an excellent potential to differentiate AD‐aMCI‐HL from CN (an AUC of 98%).

Our findings in PD‐NC versus PD‐MCI nicely align with the literature on hippocampal atrophy in PD, which is specific to PD‐MCI and PD‐dementia phases (Das et al., 2019; Mak et al., 2015; Segura et al., 2014).

We hypothesize that these findings are the reason for our finding of cued recall and associative memory impairment gradient: AD‐aMCI>PD‐MCI>PD‐NC>CN. From the point of differential reasoning, we show robust evidence that AD‐aMCI presents as a clinical unit with a pronounced deficit in cued recall even in comparison to the same stage of cognitive evolution (PD‐MCI), due to different aetiology. However, free recall measures (RAVLT‐DR and 1–5) did reach comparable levels of classification accuracy, especially in PD‐MCI versus PD‐NC; PD‐MCI versus CN and CN versus AD‐aMCI‐HL.

Also, the question of the relation between demographics and MBT measures must be addressed in comparison with previous seminal studies (Gramunt et al., 2015, 2016). As expected based on the literature for memory‐challenging tasks (Sherman et al., 2023), we replicated a medium to low negative relationship between age and most MBT outcomes (Gramunt et al., 2016). In contrast with previous reports, the relationships between education level or sex and MBT measures were non‐existent except for the MBT‐TIP, which converges with the results found in Gramunt et al. (2016). This finding can be explained by previous studies on cued recall and education, which found that with advancing age, the level of education becomes a more important predictor of memory efficiency than age (Van Der Linden et al., 1993). Our CN sample was medium to highly educated.

The current study has several limitations that must be acknowledged. First, our PD cohort has a moderate sample size. Second, only a subgroup of the AD‐aMCI (AD‐aMCI‐HL) had metabolic biomarkers available. However, both cohorts (PD and AD‐aMCI) are based on the standard clinical criteria. Regarding the absence of biomarkers in PD‐MCI, we are well aware of the rapidly changing landscape regarding the availability of biomarkers for PD diagnosis (Mastenbroek et al., 2024; Rennie et al., 2024). However, at the time of data collection, no biomarkers were available in our PD sample. Third, there is evidence of overlap between α‐synuclein pathology in PD and AD; however, the ratio of these cases in our PD sample is unknown (Desikan et al., 2015). More importantly, no CSF or imaging biomarkers were available for the PD‐MCI group, which precludes definitive exclusion of underlying AD pathology within this cohort. This absence of biomarkers may have contributed to potential heterogeneity within our diagnostic groups. Future studies should address this limitation by employing fully biomarker‐defined samples. Fourth, due to sample size, we were not able to investigate if there are memory differences in PD‐MCI subtypes (e.g. single‐ vs. multiple‐domain or amnestic vs. non‐amnestic presentation) (Jellinger, 2024; Kalbe et al., 2016; Yarnall et al., 2013). Fifth, given the differences in diagnostic criteria—where the AD‐aMCI group was diagnosed primarily based on the presence of memory impairment, while the PD‐MCI group did not require domain‐specific impairment—the between‐group differences and discriminability observed in this study may relate less to specific memory processes (e.g. associative memory and binding impairment) and more to the general presence of memory impairment, irrespective of its specific nature. Further research should incorporate PD‐MCI subtypes to strengthen the validation of these findings. In addition, memory impairment in the AD‐aMCI group was defined as a score ≥ 1.5 *SD* below the mean of age‐ and education‐adjusted norms on at least one of six memory variables. While this aligns with standard criteria (Albert et al., 2011), it may increase the risk of false positives. Future studies may benefit from applying a base‐rate approach to low scores to improve diagnostic specificity (Oltra‐Cucarella et al., 2021). Moreover, our MBT data did not include the delayed recall condition, which is a sensitive marker of memory deficits in AD‐aMCI and PD‐MCI and would result in different classification accuracies.

In conclusion, we used the MBT, a memory measure derived from translational efforts in cognitive neuroscience, to show differential cue sensitivity under controlled learning in AD‐aMCI vs. PD‐MCI. We found a prominent associative memory impairment in cued recall and binding, especially in those patients suffering from AD‐aMCI‐HL, that can be psychometrically differentiated from PD‐MCI, PD‐NC and CN. In conclusion, the MBT is less time‐consuming than RAVLT, and is a highly discriminative tool with above 90% detection potential to differentiate memory impairments not only between AD‐aMCI‐HL and CN but also between AD‐aMCI and PD‐MCI. We can conclude that the MBT is useful in clinical practice and reliably differentiates the profiles and degree of memory impairment in the most common neurodegenerative diseases.

## AUTHOR CONTRIBUTIONS

**Ondrej Bezdicek:** Conceptualization; investigation; funding acquisition; writing – original draft; methodology; validation; visualization; writing – review and editing; formal analysis; project administration; data curation; supervision; resources. **Jiří Motýl:** Conceptualization; methodology; software; formal analysis; project administration; data curation; writing – review and editing; writing – original draft; visualization; validation; supervision. **Tomáš Nikolai:** Conceptualization; investigation; funding acquisition; writing – original draft; methodology; validation; writing – review and editing; supervision. **Adéla Fendrych Mazancová:** Conceptualization; investigation; funding acquisition; writing – original draft; methodology; validation; writing – review and editing. **Jakub Hort:** Conceptualization; investigation; funding acquisition; writing – original draft; methodology; validation; writing – review and editing; supervision. **Robert Jech:** Supervision; conceptualization; investigation; funding acquisition; writing – original draft; methodology; validation; writing – review and editing. **Martin Vyhnálek:** Supervision; conceptualization; funding acquisition; writing – original draft; visualization; writing – review and editing; methodology; validation; investigation; formal analysis; data curation; resources. **Hana Horáková:** Conceptualization; investigation; funding acquisition; writing – original draft; methodology; validation; visualization; writing – review and editing; formal analysis; project administration; data curation; supervision; resources.

## FUNDING INFORMATION

This work was supported by the National Institute for Neurological Research, Czech Republic, Programme EXCELES, ID Project No. LX22NPO5107, funded by the European Union ‐ Next Generation EU, and also by the Charles University: Cooperatio Program in Neuroscience, by Ministryof Health, Czech Republic ‐ conceptual development of research organization, Motol University Hospital, Prague, Czech Republic 00064203, and by the Czech Science Foundation (GACR) registration number 22‐33968S.

## CONFLICT OF INTEREST STATEMENT

The authors have no financial interests in the Memory Binding Test and have nothing to disclose.

## Supporting information


**Data S1:** Supporting Information.

## Data Availability

The data that support the findings of this study are available on request from the corresponding author. The data are not publicly available due to privacy or ethical restrictions.
