# Supplementary material for: Differential cued recall memory impairment in mild cognitive impairment due to Alzheimer's disease versus Parkinson's disease
Source: J Neuropsychol. 2025 Aug 28;20(1):68–87. doi: 10.1111/jnp.70010 (PMC12976840; doi:10.1111/jnp.70010)

**Supplementary Material**

Supplementary Table 1

*Robust ANCOVA showing the difference between CN and AD-aMCI in MBT-TIP score for different Age levels*

| Age | n1 (CN) | n2 (AD-aMCI) | Mean Difference | SE | lower CI | upper CI | statistic | *p*-value |
| --- | --- | --- | --- | --- | --- | --- | --- | --- |
| 65 | 102 | 12 | 14.052 | 2.3603 | 6.148 | 21.957 | 5.954 | < .001 |
| 67 | 99 | 13 | 13.080 | 2.2283 | 5.880 | 20.280 | 5.870 | < .001 |
| 70 | 90 | 23 | 11.382 | 1.7697 | 6.216 | 16.547 | 6.432 | < .001 |
| 72 | 88 | 33 | 9.029 | 1.5494 | 4.718 | 13.340 | 5.828 | < .001 |
| 79 | 50 | 16 | 7.368 | 2.4765 | -0.346 | 15.078 | 2.975 | .014 |

*Note.* CN, Clinically cognitively normal controls; AD-aMCI, Alzheimer’s Disease Amnestic Mild Cognitive Impairment.

Supplementary Table 2

*Kendall’s tau-b correlation coefficients between MBT measures and other neuropsychological methods in CN and clinical samples*

|  | MBT CR List 1+2 | MBT-TIP | MBT-PIP | MBT-FR 2min |
| --- | --- | --- | --- | --- |
| MBT-TIP | 0.765*** |  |  |  |
| MBT-PIP | 0.803*** | 0.946*** |  |  |
| MBT-FR 2min | 0.585*** | 0.603*** | 0.628*** |  |
| MMSE | 0.130* | 0.123 | 0.192* | 0.157* |
| TMT-A | -0.128* | -0.159* | -0.112 | -0.171* |
| TMT-B | -0.264*** | -0.251*** | -0.190* | -0.277*** |
| PST-D | -0.066 | -0.131* | -0.160 | -0.108 |
| PST-W | -0.084 | -0.196** | -0.288*** | -0.156* |
| PST-C | -0.216*** | -0.185** | -0.204* | -0.261*** |
| RAVLT 1-5 | 0.323*** | 0.337*** | 0.371*** | 0.412*** |
| RAVLT DR 30 min | 0.437*** | 0.420*** | 0.469*** | 0.471*** |

*Note.* MBT, Memory Binding Test; CR L1+L2, Number of items cued recalled from List 1 and List 2 on the MBT; TIP, Total number of items cued recalled in the Paired condition on the MBT; PIP, The number of pairs cued recalled in the paired condition of MBT; FR 2 min, Total number of items recalled in the 2 minutes free recall condition on the MBT; MMSE, Mini Mental State Examination; TMT, Trail Making Test; PST, Prague Stroop Test; PST-D (Dots, Naming Colors); PST-W (Words, Weak Interference); PST-C (Colors; Interference condition); RAVLT, Rey Auditory Verbal Learning Test; (RAVLT 1-5, Immediate Recall in Trials 1-5; RAVLT DR 30 min, Delayed Free Recall Condition after 30 minutes). * *p* < .05, ** *p* < .01, *** *p* < .001

Supplementary Figure 1

*ROC Curve, CN vs. AD-aMCI*


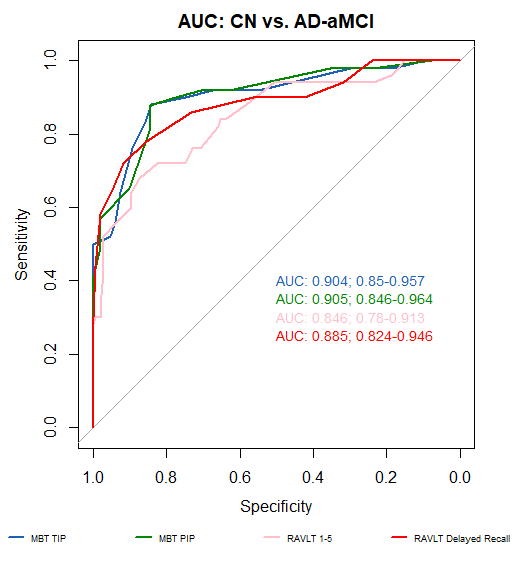


*Note.* CN, Clinically cognitively normal controls; AD-aMCI, Amnestic Mild Cognitive Impairment due to Alzheimer’s Disease. AUC with a 95% confidence interval.

Supplementary Figure 2

*ROC Curve, PD-MCI vs. AD-aMCI*


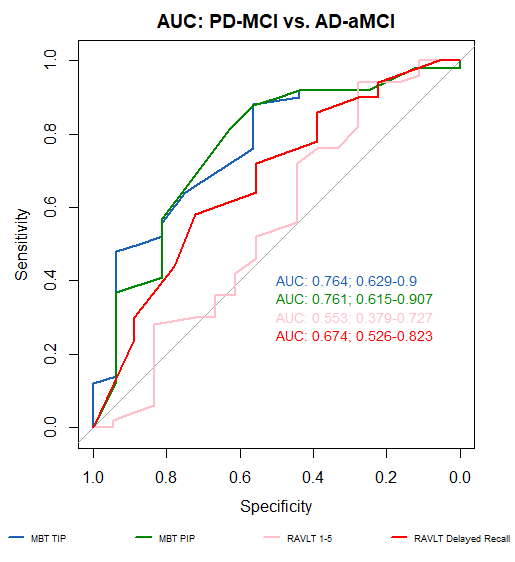


*Note.* PD-MCI, Parkinson’s Disease Mild Cognitive Impairment; AD-aMCI, Amnestic Mild Cognitive Impairment due to Alzheimer’s Disease. AUC with a 95% confidence interval.

Supplementary Figure 3

*ROC Curve, CN vs. PD-MCI*


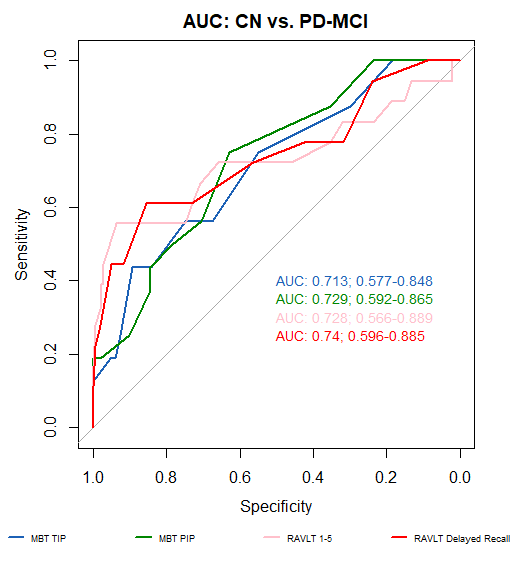


*Note.* CN, Clinically cognitively normal controls; PD-MCI, Parkinson’s Disease Mild Cognitive Impairment. AUC with a 95% confidence interval.

Supplementary Figure 4

*ROC Curve, PD-NC vs. PD-MCI*


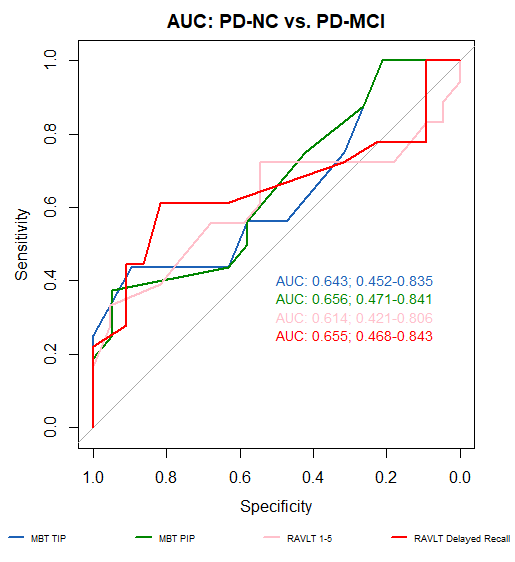


*Note.* PD-NC, Parkinson’s Disease Normal Cognition; PD-MCI, Parkinson’s Disease Mild Cognitive Impairment. AUC with a 95% confidence interval.

Supplementary Figure 5

*ROC Curve, PD-MCI vs. AD-aMCI-HL*


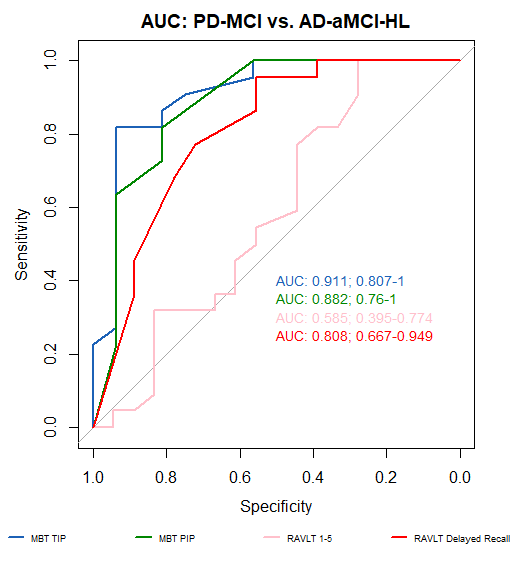


*Note.* PD-MCI, Parkinson’s Disease Mild Cognitive Impairment; AD-aMCI-HL, Amnestic Mild Cognitive Impairment due to Alzheimer’s Disease – subgroup of patients with positive biomarkers for β amyloid.

Supplementary Figure 6

*ROC Curve, CN vs. AD-aMCI-HL*


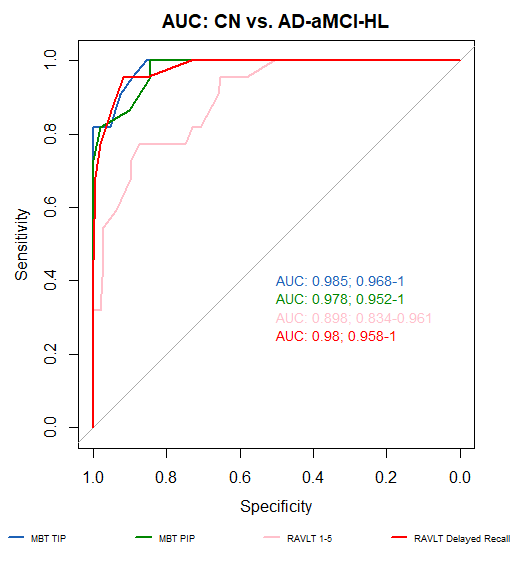


*Note.* CN, Clinically cognitively normal controls; AD-aMCI-HL, Amnestic Mild Cognitive Impairment due to Alzheimer’s Disease – subgroup of patients with positive biomarkers for β amyloid.

Supplementary Table 3

*Discriminative validity CN vs. AD-aMCI, MBT-TIP Sensitivity and Specificity*

| Cut-Off CN vs. AD-aMCI | Specificity | Sensitivity | Youden Index |
| --- | --- | --- | --- |
| 27.5 | 0.675 | 0.920 | 1.595 |
| 26.5 | 0.748 | 0.900 | 1.648 |
| 25.5 | 0.841 | 0.880 | 1.721 |
| 24.5 | 0.854 | 0.840 | 1.694 |
| 23.5 | 0.894 | 0.760 | 1.654 |
| 22.5 | 0.927 | 0.640 | 1.567 |
| 21.5 | 0.940 | 0.560 | 1.500 |

*Note*. MBT-TIP, the total number of items cued recalled in the paired condition of MBT.

Supplementary Table 4

*Discriminative validity CN vs. AD-aMCI, MBT-PIP Sensitivity and Specificity*

| Cut-Off CN vs. AD-aMCI | Specificity | Sensitivity | Youden Index |
| --- | --- | --- | --- |
| 12.5 | 0.627 | 0.918 | 1.546 |
| 11.5 | 0.706 | 0.918 | 1.624 |
| 10.5 | 0.784 | 0.898 | 1.682 |
| 9.5 | 0.843 | 0.878 | 1.721 |
| 8.5 | 0.843 | 0.816 | 1.659 |
| 7.5 | 0.902 | 0.653 | 1.555 |
| 6.5 | 0.980 | 0.571 | 1.552 |

*Note*. MBT-PIP, the number of pairs cued recalled in the paired condition of MBT.

Supplementary Table 5

*Percentile range of the normative sample on MBT-TIP and MBT-PIP*

| MBT-TIP |  | MBT-PIP |  |
| --- | --- | --- | --- |
| N = 151 Age M = 62.57 (±9.74; 38 - 85) M = 27.96 (±3.03; 20 - 32) | | N = 51 Age M = 66.02 (±7.88; 46 - 85) M = 12.33 (±2.76; 5 - 16) | |
| Raw Score | Percentile | Raw Score | Percentile |
| 32 | 100 | 16 | 100 |
| 31 | 91 | 15 | 92 |
| 30 | 82 | 14 | 77 |
| 29 | 70 | 13 | 65 |
| 28 | 45 | 12 | 37 |
| 27 | 33 | 11 | 29 |
| 26 | 25 | 10 | 22 |
| 25 | 16 | 9 | 16 |
| 24 | 15 | 8 | 16 |
| 23 | 11 | 7 | 10 |
| 22 | 7 | 6 | 2 |
| 21 | 6 | 5 | 2 |
| 20 | 5 |  |  |

*Note*: Percentiles were estimated as quantiles and rounded to an integer.
MBT-TIP: total number of items cued recalled in the paired condition of MBT; MBT-PIP: the number of pairs cued recalled in the paired condition of MBT.

Supplementary Table 6

*Demographic characteristics and test scores of clinically cognitively normal controls and patients’ groups, with AD-aMCI group divided based on availability of biomarkers.*

|  | CN | PD-NC | PD-MCI | AD-aMCI | AD-aMCI-HL | *p*-value | Effect size |
| --- | --- | --- | --- | --- | --- | --- | --- |
| N | 161 | 22 | 18 | 28 | 22 |  |  |
| Sex (F/M) | 85/76 | 10/12 | 8/10 | 11/17 | 13/9 | 0.577^1^ | 0.107^3^ |
| Age (M, SD, min-max) | 62.63 (±9.76; 38.00-85.00) | 63.82 (±7.40; 45.00-81.00) | 62.78 (±9.91; 37.00-82.00) | 72.87 (±8.61; 51.00-88.00) | 71.50 (±4.37; 62.00-78.00) | <0.001^2, c,d,f,g,h,i^ | 0.171^4^ |
| Education (years, M, SD, min-max) | 14.50 (±2.74; 9.00-25.00) | 14.80 (±2.86; 11.00-21.00) | 13.11 (±2.89; 8.00-19.00) | 16.39 (±3.06; 9.00-20.00) | 15.27 (±3.07; 8.00-19.00) | <0.001^2, c,h^ | 0.077^4^ |
| PD duration (years) | - | 11.12 (±5.61; 4.00-26.00) | 12.86 (±2.85; 9.00-18.00) | - | - | 0.200^2^ | 0.007^4^ |
| UPDRS-III “on” state | - | 17.00 (±10.75; 4.00-39.00) | 18.93 (±8.96; 3.00-32.00) | - | - | 0.519^2^ | 0.002^4^ |
| Hoehn/Yahr stage | - | 2.00 (±0.48; 1.50-3.00) | 2.04 (±0.60; 1.00-3.00) | - | - | 0.744^2^ | 0.000^4^ |
| L-Dopa Equivalent | - | 1310.86 (±747.27; 0.00-2828.80) | 1457.27 (±603.77; 450.00-2731.00) | - | - | 0.339^2^ | 0.004^4^ |
| MMSE* | 28.64 (±1.14; 25.00-30.00) | 28.11 (±1.49; 25.00-30.00) | 26.19 (±3.17; 17.00-30.00) | 28.04 (±1.43; 25.00-30.00) | 27.32 (±1.39; 25.00-30.00) | <0.001^5, b,d^ | 0.123^6^ |
| TMT-A** | 41.54 (±14.07; 20.00-88.00) | 40.27 (±9.04; 20.00-55.00) | 69.56 (±28.43; 34.00-131.00) | 48.47 (±19.12; 25.00-82.40) | 43.32 (±13.11; 26.73-70.80) | <0.001^5, b,e,h,i^ | 0.134^6^ |
| TMT-B** | 93.19 (±38.23; 38.00-290.00) | 100.50 (±26.89; 52.00-164.00) | 228.78 (±104.06; 91.00-416.00) | 156.35 (±86.87; 58.30-315.00) | 125.88 (±67.27; 48.80-301.00) | <0.001^5, b,c,e,h,i^ | 0.277^6^ |
| PST-D** | 14.88 (±3.87; 10.00-28.00) | 12.73 (±2.07; 9.00-17.00) | 15.00 (±3.77; 10.00-25.00) | 14.84 (±4.20; 10.39-31.97) | 15.30 (±4.28; 11.30-31.30) | 0.063^5^ | 0.036^6^ |
| PST-W** | 19.41 (±6.79; 11.00-46.20) | 15.18 (±2.48; 11.00-20.00) | 20.61 (±5.00; 14.00-29.00) | 18.46 (±4.19; 12.57-29.62) | 22.82 (±21.85; 13.70-120.00) | 0.009^5, a,e,g^ | 0.053^6^ |
| PST-C** | 28.50 (±8.20; 16.40-51.00) | 29.41 (±8.10; 18.00-57.00) | 45.22 (±23.58; 18.00-110.00) | 39.00 (±11.86; 20.10-63.72) | 36.70 (±14.97; 18.00-77.90) | <0.001^5, b,c,e^ | 0.131^6^ |
| RAVLT T1-5 | 47.38 (±8.24; 28.00-74.00) | 41.41 (±7.79; 28.00-56.00) | 37.50 (±13.25; 15.00-64.00) | 36.18 (±9.86; 20.00-55.00) | 33.32 (±7.60; 18.00-47.00) | <0.001^5, a,b,c,d^ | 0.228^6^ |
| RAVLT-DR | 9.14 (±2.70; 2.00-15.00) | 8.00 (±2.60; 3.00-14.00) | 5.94 (±3.86; 0.00-12.00) | 5.07 (±3.58; 0.00-11.00) | 1.95 (±2.03; 0.00-7.00) | <0.001^5, b,c,d,f,g,,i,j^ | 0.356^6^ |
| MBT CR L1* | 15.49 (±0.86; 12.00-16.00) | 15.00 (±1.15; 13.00-16.00) | 14.56 (±1.59; 10.00-16.00) | 13.96 (±2.38; 7.00-16.00) | 10.32 (±3.11; 5.00-15.00) | <0.001^5, b,c,d,g,i,j^ | 0.375^6^ |
| MBT CR L2* | 13.29 (±2.20; 7.00-16.00) | 12.89 (±1.82; 9.00-16.00) | 11.12 (±3.14; 4.00-16.00) | 9.43 (±3.43; 2.00-14.00) | 6.41 (±3.00; 2.00-13.00) | <0.001^5, b,c,d,g,i^ | 0.234^6^ |
| MBT CR L1+L2* | 28.78 (±2.62; 22.00-32.00) | 27.89 (±2.23; 24.00-31.00) | 25.69 (±4.53; 14.00-32.00) | 23.39 (±5.16; 12.00-30.00) | 16.73 (±5.24; 9.00-28.00) | <0.001^5, b,c,d,i,j^ | 0.294^6^ |
| MBT-TIP* | 27.96 (±3.03; 20.00-32.00) | 27.16 (±2.81; 23.00-31.00) | 24.56 (±5.45; 9.00-30.00) | 21.68 (±5.94; 8.00-31.00) | 13.36 (±5.82; 4.00-24.00) | <0.001^5, b,c,d,f,g,i,j^ | 0.332^6^ |
| MBT-PIP | 12.33 (±2.76; 5.00-16.00) | 11.58 (±2.61; 7.00-15.00) | 9.44 (±4.07; 0.00-14.00) | 7.48 (±3.63; 0.00-15.00) | 3.09 (±2.86; 0.00-9.00) | <0.001^5, b,c,d,f,g,i,j^ | 0.507^6^ |
| MBT-FR 2 min | 17.57 (±4.55; 6.00-28.00) | 15.47 (±3.41; 8.00-24.00) | 10.38 (±6.66; 0.00-20.00) | 10.07 (±5.51; 0.00-18.00) | 4.77 (±4.23; 0.00-13.00) | <0.001^5, b,c,d,e,g,j^ | 0.432^6^ |

*Note*. CN, clinically cognitively normal controls; PD-NC, Parkinson’s Disease Normal Cognition; PD-MCI, Parkinson’s Disease Mild Cognitive Impairment; AD-aMCI, Alzheimer’s Disease Amnestic Mild Cognitive Impairment without biomarkers for β amyloid available; AD-aMCI-HL, Amnestic Mild Cognitive Impairment due to Alzheimer’s Disease – subgroup of patients with positive biomarkers for β amyloid.; MMSE, Mini Mental State Examination; TMT, Trail Making Test; PST, Prague Stroop Test; (PST-D (Dots, Naming Colors); PST-W (Words, Weak Interference); PST-C (Colors; Interference condition); RAVLT, Rey Auditory Verbal Learning Test (RAVLT T1-5 is the sum of all correct responses given over the five consecutive trials: (T1 + T2 + T3 + T4 + T5); RAVLT-DR (30 min): The total number of correct words recalled on the delayed recall); MBT, Memory Binding Test; CR-L1, Number of items cued recalled from List 1 on the MBT; CR-L2, Number of items cued recalled from List 2 on the MBT; CR L1+L2, Number of items cued recalled from List 1 and List 2 on the MBT; TIP, Total number of items cued recalled in the Paired condition on the MBT; PIP, The number of pairs cued recalled in the paired condition of MBT; FR 2 min, Total number of Items recalled in the 2 minutes Free recall condition on the MBT; *The variable was log10 transformed for the purposes of ANCOVA analyses, **The variable was reflected and log10 transformed for the purposes of ANCOVA analyses; ^1^Chi-Squared Test, ^2^Kruskal-Wallis H-test, ^3^Crammer’s V, ^4^ε^2^, ^5^ANCOVA with Age and Years of Education as covariates, ^6^Partial η^2^; Significant between-group differences in post-hoc tests using Tukey's HSD (honestly significant difference) test: ^a^CN vs. PD-NC, ^b^CN vs. PD-MCI, ^c^CN vs. AD-aMCI, ^d^CN vs. AD-aMCI-HL, ^e^PD-NC vs. PD-MCI, ^f^PD-NC vs. AD-aMCI, ^g^PD-NC vs. AD-aMCI-HL, ^h^PD-MCI vs. AD-aMCI, ^i^PD-MCI vs. AD-aMCI-HL, ^j^AD-aMCI vs. AD-aMCI-HL.

**Matched Sample Analysis**

In the matched sample analysis, we included 31 healthy controls (HC) and 50 AD-aMCI patients. The mean age of the AD-aMCI group was 72.27 years, and the mean age of the matched HC group was 71.72 years, with a mean age difference of 0.55 years. We then performed ROC analyses to compare the diagnostic performance of selected tests between the matched HC and AD-aMCI groups.

For the comparison between matched HC (n=31) and AD-aMCI (n=50): The area under the curve (AUC) for MBT TIP was 0.9223 (95% CI: 0.8647–0.9798). The AUC for RAVLT 1-5 was 0.8477 (95% CI: 0.7667–0.9288). The AUC for RAVLT Delayed Recall was 0.8997 (95% CI: 0.8365–0.9629).

Additionally, we performed a comparison between a subset of the matched HC group (n=31) and AD-aMCI patients with amyloid confirmation (n=22): For the comparison between matched HC (n=31) and AD-aMCI with amyloid confirmation (n=22): The AUC for MBT TIP was 0.9949 (95% CI: 0.9851–1.0000). The AUC for RAVLT 1-5 was 0.9003 (95% CI: 0.8183–0.9823). The AUC for RAVLT Delayed Recall was 0.9875 (95% CI: 0.9647–1.0000).

These results demonstrate that even after age matching, the diagnostic performance of the tests remains strong, particularly for MBT TIP and RAVLT Delayed Recall when comparing matched HC to AD-aMCI. The results remain comparable to the original analysis, only the discriminative performance of the selected test generally improves after matching.

We hope that these additional analyses address the reviewer's concerns and provide further support for our findings. A lot of information regarding this question can also be found in Markova et al. (2023).


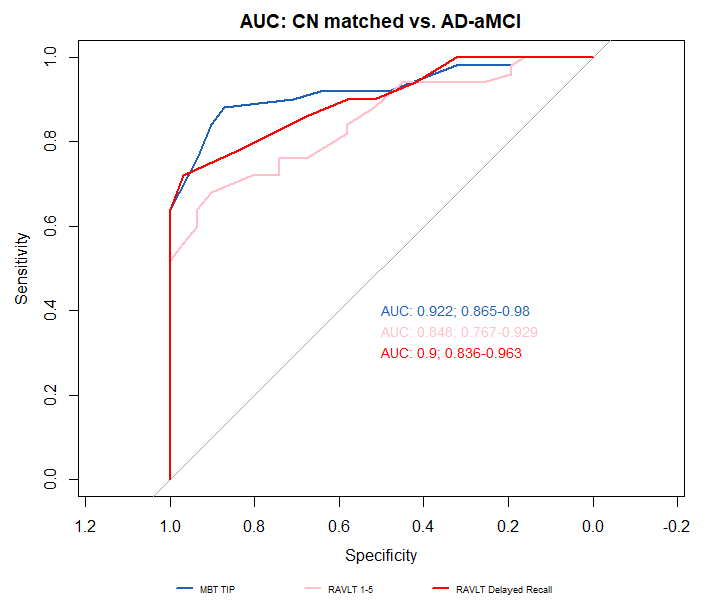


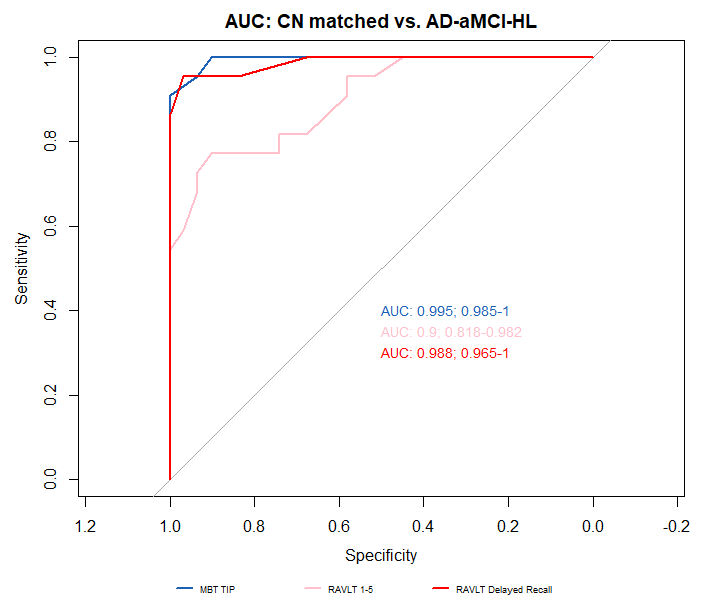

Supplement: Supplementary file 1 — Data S1: Supporting Information. [file JNP-20-68-s001.docx]
